# Early-Onset Gastrointestinal Malignancies: An Investigation into a Rising Concern

**DOI:** 10.3390/cancers16081553

**Published:** 2024-04-18

**Authors:** Aayush Vishwanath, Shreyas Krishna, Albert P. Manudhane, Phil A. Hart, Somashekar G. Krishna

**Affiliations:** 1 Department of Neuroscience, The Ohio State University, Columbus, OH 43210, USA; vishwanath.19@osu.edu; 2 Division of Gastroenterology, Hepatology and Nutrition, The Ohio State University, Columbus, OH 43210, USA; krishna.111@osu.edu (S.K.); albert.manudhane@osumc.edu (A.P.M.);

**Keywords:** early-onset gastrointestinal malignancies, young adult cancer, early-onset colorectal cancer, early-onset esophageal cancer, esophageal adenocarcinoma, esophageal squamous cell carcinoma, early-onset gastric cancer, early-onset pancreatic cancer, early-onset liver cancer, early-onset biliary malignancy

## Abstract

**Simple Summary:**

In recent decades, there has been an increase in gastrointestinal malignancies diagnosed in individuals below the age of 50. Improved recognition of risk factors for these cancers may lead to prevention or earlier screening, which can reduce disease morbidity and mortality. This study aims to comprehensively review the characteristics, genetic factors, and environmental risks of early-onset gastrointestinal and liver cancers. Incidence trends are highlighted for these malignancies, as are screening guidelines. Although we describe many well-established risk factors for early-onset gastrointestinal malignancies, future population-based studies may help identify additional risk factors and shed light on improved screening strategies or early detection techniques.

**Abstract:**

There is growing recognition of early-onset gastrointestinal (GI) malignancies in young adults < 50 years of age. While much of the literature has emphasized colorectal cancer, these also include esophageal, gastric, liver, pancreatic, and biliary tract malignancies. Various factors, including lifestyle, hereditary, and environmental elements, have been proposed to explain the rising incidence of GI malignancies in the younger population. This review aims to provide an overview of the recent literature, including global trends and information regarding genetic and environmental risk factors.

## 1. Introduction

The definition of a young adult, typically used to assess cancer incidence, encompasses individuals below 50 years of age [1]. There has been a rise in the global incidence of malignancies among young adults reported internationally—most frequently gastrointestinal cancers, breast cancer, endometrial cancer, kidney cancer, bone marrow cancer, head and neck cancers, and prostate cancer [1]. Herein, we will describe the increase in, characteristics of, and screening recommendations for early-onset gastrointestinal malignancies, which include esophageal, gastric, colorectal, liver, pancreatic, and biliary tract cancer (Table 1, Table 2 and Table 3). A comprehensive review of the literature was performed.

## 2. Esophageal Cancer

### 2.1. Esophageal Cancer: Introduction

According to a GLOBCAN database analysis, there were more than 604,000 new cases of esophageal cancer and approximately 544,000 deaths attributed to this disease in the year 2020, making it the eighth most common cancer worldwide [2]. Incidence and mortality rates were the highest in Eastern Asia and Southern and Eastern Africa, and the malignancy is 2–3-fold higher in men than women [2]. In contrast, the lowest incidence and mortality rates are found in Western Africa and Central America [2].

### 2.2. Esophageal Cancer (EAC and SCC): Global and US Incidence

Squamous cell carcinoma (SCC) and esophageal adenocarcinoma (EAC) are the two primary types of esophageal malignancies, each associated with distinct risk profiles. Historically, SCC has been the most common type of esophageal cancer worldwide; however, over the past three decades, there has been a significant increase in the incidence of EAC, making it the predominant form of esophageal malignancy in Westernized countries [2]. While SCC originates from the stratified squamous epithelial lining of the esophagus, EAC develops in the columnar glandular cells that replace the squamous epithelium (from the precursor Barrett’s esophagus). Barrett’s esophagus (BE) is characterized by distinct and identifiable metaplastic salmon-colored “tongues” that extend from the gastroesophageal junction.

Demographically, SCC and EAC exhibit substantial differences and nuances. These variations in demographic patterns emphasize the distinct characteristics and complexities associated with each type of esophageal cancer. In the United States (US), the incidence of SCC is notably higher in the Black population compared to the White population. Remarkably, the incidence of SCC has decreased across all racial and ethnic groups, with a particularly higher rate of decrease observed in the Black population [16]. EAC exhibits a higher incidence in the White population than in the Black population. The racial differences in the incidence of EAC are partly attributable to the increased risk of BE in the White population. The prevalence of BE is higher in men compared to women (2:1 ratio); thus, the progression to EAC is also greater in men [12].

### 2.3. Esophageal Cancer: Incidence in Young Adults

According to an analysis of the US Surveillance, Epidemiology, and End Results (SEER) database, the annual incidence of EAC in individuals under 50 years of age increased more than three-fold, from 0.08 per 100,000 to 0.27 per 100,000, between 1975 and 2015 [103]. In another analysis from Australia, a cohort of young-onset individuals exhibited a substantial upward trend in the incidence rates of EAC. The incidence rate ratio (IRR) for the period 2010–2017 compared to 1990–1999 was 2.60 (95% CI 1.35–5.03) [104].

An analysis of the National Cancer Database revealed a plateau of or a slight decline in total EAC rates from 2004 to 2015; early-onset EAC incidence in the period of 2004–2006 was notably 4.2% higher than that observed in 2013–2015 [17]. A recent SEER analysis revealed the stabilization of or a decline in the overall incidence rates of EAC; for the 50–54-year-old cohort, the annual percent change (APC) in EAC rates from 2000 to 2019 was (−)1.15. In the 45–49-year-old cohort, from 1992 to 2019 (including an initial increase from 1992 to 2000), the APC was (−)0.13. These figures indicate that there was a stabilization of or potential decrease in the rates of EAC within the 49–54-year-old age group up until 2019 [24]. A broader analysis of the SEER database, covering a wider variety of age groups, starting at 20 years of age and including all histologic types of esophageal malignancies, also observed a decline in esophageal cancer rates for the 18–49-year-old age cohort from 2004 to 2013. The study reported an average annual percent change of (−)1.8% in incidence rates during this period [25].

Esophageal cancer, encompassing both SCC and EAC, carries a grim prognosis and outcome for the majority of its cases, as the overall five-year survival rate is 15% to 20% [105]. Patients typically have advanced disease at the time of diagnosis, which contributes to the lower survival rate. However, there has been a recent increase in 1-year survival for EAC and SCC in high-income countries due to advances in treatment [106]. 

### 2.4. Esophageal Cancer: Risk Factors for Early-Onset Disease

SCC and EAC both have distinct risk profiles; therefore, each histologic type of EC was observed to have different genetic and environmental factors. 

#### 2.4.1. Squamous Cell Carcinoma Risk Factors

There is a paucity of research investigating early-onset SCC. A recent study from Tanzania identified multiple exposures as risk factors for early-onset esophageal SCC, including infrequent teeth cleaning, exposure to secondhand tobacco smoke, and pest infestation of grain and/or nuts [42]. Lower socioeconomic status, a family history of SCC, tobacco smoking, home-brewed alcohol consumption, home storage of grain and/or nuts, and use of firewood for cooking were associated with SCC risk in the older age group but not in the younger population. Hot beverage intake was found to be associated with an increased risk of SCC regardless of age.

#### 2.4.2. Esophageal Adenocarcinoma Risk Factors

One California study found a 56% increase in early-onset EAC in obese patients and a striking 166% increase in morbidly obese patients [40]. Another US study with 335 EAC patients confirmed the relationship between obesity and early-onset EAC [41]. Other known risk factors, such as sex, gastric acid reflux symptoms, and smoking, did not show significant differences between early- and late-onset patients [41]. In a study from the Netherlands, heredity was implicated in some cases of early-onset EAC, with familial BE accounting for 7% of cases. TP53 and P16 mutations were common in both early-onset and conventional EACs, but additional mutations in different genes were found exclusively in early-onset cases [60].

### 2.5. Esophageal Cancer: Improvements in Detection Measures and Screening Guidelines

Guidelines recommend a single screening endoscopy for patients with chronic reflux symptoms and three or more additional risk factors for BE. These risk factors include male sex, age > 50 years, White race, tobacco smoking, obesity, and a family history of BE or EAC in a first-degree relative [78]. If the screening esophagogastroduodenoscopy (EGD) reveals non-dysplastic BE, surveillance every 3–5 years is recommended. However, if low-grade dysplasia is detected, then annual endoscopy is recommended until two consecutive endoscopies show no evidence of dysplasia [78]. 

Advancements in screening for SCC are limited due to the complexity of the disease’s precursors. Even in countries like China, where SCC is highly prevalent, population-based screening is not currently recommended, as no screening test has been proven to lower mortality in average-risk individuals. Further studies are necessary to assess the cost-effectiveness and feasibility of screening for SCC [95].

While EGD is the primary method for detecting BE and SCC, there are viable and cost-effective alternatives available. Unsedated transnasal endoscopy (uTNE) involves using an ultra-thin endoscope introduced through the nasal cavity to examine the esophagus and stomach. uTNE has demonstrated similar sensitivity and specificity to EGD for detecting BE and comes with the advantage of lower cost due to the lack of sedation and need for intensive monitoring [79]. Another cost-effective alternative is the cytosponge, which consists of a gelatin-coated sponge attached to a string and is swallowed by the patient. The cytosponge expands within the esophagus to collect cytology specimens and has comparable sensitivity and specificity to EGD for BE detection (73% and 94%, respectively). With the emergence of genomic or molecular markers for BE and EAC, there is a possibility that the cytosponge will be incorporated into the previously discussed surveillance protocols for BE [80]. Additionally, a UK study indicated that the cytosponge was more comfortable, practical, and economical than endoscopy [107].

## 3. Gastric Cancer

### 3.1. Gastric Cancer: Introduction

In 2020, gastric cancer was ranked as the fifth most common malignancy in the world and the second most lethal, with men experiencing a two-fold greater incidence rate [8,13]. The incidence of gastric cancer was found to be the greatest in Asia, followed by the Caribbean, Europe, and Oceania. The lowest incidence occurred in North America and Africa. Hispanic and Black patients are disproportionately affected by early-onset gastric cancer and are often faced with a worse prognosis. This disparity could be related to delays in diagnosis and reduced access to optimal care [17]. A 2008–2014 retrospective cohort study confirmed that lower socioeconomic status and racial and ethnic minorities face a higher gastric cancer risk than non-Hispanic White individuals [13].

Approximately 95% of all gastric malignancies are adenocarcinomas, and the remaining 5% of cases mainly comprise gastrointestinal stromal tumors, neuroendocrine tumors, and lymphomas [3]. Gastric adenocarcinomas are commonly categorized anatomically as either gastric cardia or non-cardia cancer. These classifications have distinct epidemiological features; for instance, non-cardia gastric cancer is more common in East Asian and Latin American countries, while gastric cardia cancer is more common in Western Europe and North America [4]. 

Recent findings also predict an overall increase in both the annual incidence and mortality rates of gastric cancer by the year 2040 to an estimated 1.8 million new cases and 1.3 million deaths, with gastric cancer increasingly found in the young [7]. One study estimates that 30% of newly diagnosed gastric cancers are early-onset [26]. Another international population-based cohort analyzing data from 1980 to 2018 found the incidence of gastric cancer to decrease in most regions among older adults but increase in those under the age of 40 [27]. These findings of increased early-onset gastric cancer incidence were confirmed by a Chinese study that analyzed trends from 1990 to 2019 [28]. 

### 3.2. Gastric Cancer: Risk Factors for Early-Onset Disease

Recent research suggests a large genetic component in early-onset gastric carcinogenesis [108,109]. Approximately 10% of those diagnosed with early-onset gastric cancer have a family history of the disease [13]. *CDH1* germline mutations can lead to abnormal encoding of the E-cadherin protein, which may result in hereditary diffuse gastric cancer associated with worse overall survival [61,62,63]. Genetic mutations such as the *CDH1* germline mutation or *hMLH1* germline mutations make up 2–3% of early-onset gastric cancer cases in North America [64]. A study evaluating the risk of developing gastric cancer in patients with Lynch syndrome found that the cumulative lifetime risk ranges from 3% to 39% across all mutations (*MLH1*, *MSH2*, *MSH6*, *PMS2*, and *EPCAM*) [65,110]. Peutz–Jeghers syndrome (PJS) is another hereditary risk factor for early-onset gastric cancer [66]. Additionally, one Japanese study evaluating the risk of malignant tumors as a result of juvenile polyposis syndrome (JPS) found that, in a sample of 171 cases, the lifetime risk of gastric cancer was as high as 73.0% [67]. 

The remaining 90% of those diagnosed with early-onset gastric cancer who lack a family history of the disease may have environmental triggers, such as *H. Pylori*, especially seen with the cagA strain contributing to non-cardia cancers [43,44,45,111]. Atrophic gastritis occurring due to *H. Pylori* can also predispose those affected to early-onset gastric cancer [43,46,47,48,112,113]. Two recent studies found a strong positive association between obesity and early-onset gastric cancer [40,49]. A BRFSS (Behavioral Risk Factor Surveillance Survey) analysis conducted to explore differences in the rates of potential risk of both early-onset gastric cancer and traditional gastric cancer found a strong positive association between heavy drinking and the risk of both early-onset gastric cancer and traditional gastric cancer, though smoking did not show any risk association [50]. 

### 3.3. Gastric Cancer: Screening and Current Detection Measures

Currently, there are no guidelines recommending routine screening for gastric cancer in the United States, though recent research recommends the consideration of EGD screening for high-risk populations with a family history of or genetic predisposition to gastric cancer [81,82]. The American College of Gastroenterology (ACG) has outlined screening recommendations for those with hereditary gastric cancer syndromes. The ACG recommends individuals with or at risk for Lynch syndrome to screen for gastric cancer by EGD with gastric biopsy beginning at ages 30–35, patients with symptoms or a family history of PJS to undergo genetic evaluation, and individuals with symptoms of PJS to also undergo a genetic evaluation [114]. In addition to EGD screening, radiographic gastric cancer screening programs are also used in countries such as Japan [83]. The implementation of screening via EGD in East Asian countries such as Japan and Korea has proven effective in reducing gastric cancer-related mortality while also being cost-efficient for those regions, based on their local incidence rates. Japanese guidelines recommend screening every 2–3 years for individuals above the age of 50. Koreans above the age of 40 are recommended for screening every 2 years [82,96,97,98]. 

## 4. Liver and Biliary Tract Malignancies

### 4.1. Liver and Biliary Tract Malignancies: Introduction

Primary liver cancer encompasses hepatocellular carcinoma (HCC), which accounts for 75% to 85% of cases, and intrahepatic cholangiocarcinoma (ICC), constituting 10% to 15% of cases, alongside other infrequent and rare types. The main risk factor for HCC is the chronic hepatitis B virus (HBV), which confers a nearly 100-fold risk to chronic carriers [115]. ICC is a malignancy involving the lining of the bile ducts, sometimes also showing perineural and lymphovascular invasion. 

According to a GLOBOCAN study in 2020, primary liver cancer ranks as the sixth most commonly diagnosed cancer and the third leading cause of cancer-related deaths worldwide, accounting for approximately 906,000 new cases and 830,000 deaths [8]. A meta-analysis encompassing a pooled analysis of the annual percent change in HCC and ICC incidence rates between 2008 and 2019 yielded values of +2.6 and +4.3, respectively. Subgroup analyses revealed increasing trends in primary liver cancer cases within the North America/Europe/Australia region (which was previously considered a region of relatively low incidence), while contrasting trends of a decrease and stability were noted in Asia (traditionally considered a high-incidence region) [9]. Furthermore, the rates of both incidence and mortality exhibited a consistent pattern of being 2 to 3 times higher in men compared to women across most regions [8]. 

In a study of SEER data, primary liver cancer incidence and death rates were notably elevated among American Indian/Alaska Natives, with figures reaching 15.2 and 11.9 per 100,000, respectively. These rates were more than double those recorded for non-Hispanic White patients, which stood at 6.3 and 5.5 per 100,000, respectively. Black, Asian American/Pacific Islander, and Hispanic patients exhibited higher incidence and mortality rates in comparison to White patients. Increasing trends in incidence over time were observed for all of these racial groups, except for American Indian/Alaska Natives and Asian American/Pacific Islanders [18]. A study examining the regional incidence of HCC and ICC using data from GLOBOCAN’s 2018 estimates found that the age-standardized incidence rates of HCC were highest in Eastern Asia, Northern Africa, and Eastern Europe, while the lowest rates were observed in South-Central Asia [21]. For ICC, the greatest age-standardized incidence rates were reported in South-Eastern Asia, Eastern Asia, and Northern Europe, with the lowest rates also being in South-Central Asia [21].

A study conducted in the United States revealed that the incidence of HCC was highest among Asian American/Pacific Islanders, with Black, Hispanic, and non-Hispanic White patients, listed in descending order. However, the same study identified a noteworthy trend in HCC incidence, with Hispanics experiencing the highest percentage increase, while Asians saw a decline in incidence [116]. 

In another United States study focused on ICC, it was observed that the Asian American/Pacific Islander population had the highest incidence rate at 1.5 per 100,000, followed by Hispanic at 1.18 per 100,000, American Indian/Alaska Native at 0.88 per 100,000, White at 0.88 per 100,000, and Black at 0.77 per 100,000 [117]. 

### 4.2. Liver and Biliary Tract Malignancies: Incidence Trends in Young Adults

A SEER analysis revealed that the incidence of HCC was decreasing in younger and middle-aged (<60 years) adults in the US, irrespective of sex, race, or ethnicity. Specifically, the incidence for the 40–49 cohort exhibited a substantial decrease (APC: −12.2%) from 2009 to 2015 [29]. Another more recent (2010 to 2019) SEER analysis also observed a decrease (APC: −4.67%, 95% CI −5.7, −3.6) in HCC rates among young adults [30]. 

Trends in ICC incidence are opposite to those of HCC. A US population study found that ICC incidence rates increased among the 18–44 age cohort, with an estimated APC of +3.3% from 1999 to 2013 [31]. A SEER analysis conducted from 2010 to 2019 recorded an APC of +8.12% for early-onset ICC, indicating one of the fastest-growing incidence rates among gastrointestinal cancers in early-onset individuals [30]. 

### 4.3. Liver and Biliary Tract Malignancies: Risk Factors for HCC and ICC

There is limited understanding of genetic risk factors for HCC in young adults. A family history of HCC is associated with an increased risk of early-onset HCC, and this increase is greater in HBV carriers [68,69]. The other roughly 90% of early-onset HCC is attributed to environmental factors, including chronic infection with HBV. Smoking has been proven to be associated with early- but not late-onset HCC, and cirrhosis has been identified to have a lower incidence among early-onset than late-onset patients [51,52]. Metabolic dysfunction-associated fatty liver disease associated with obesity may contribute to early-onset HCC [1]. 

There is also a dearth of research involving genetics and family history leading to early-onset ICC. Caroli’s disease—characterized by cystic dilations of the intrahepatic bile ducts—is inherited in an autosomal recessive manner, with ICC occurring in up to 7% of affected individuals [70]. Other established risk factors for ICC include parasitic infections, hepatolithiasis, and primary sclerosing cholangitis (PSC) [53]. In Western populations, especially among younger individuals, the largest risk factor for ICC—primary PSC—is linked to a substantial 400-fold elevation in the risk of developing ICC [54]. The recent increase in PSC incidence among Westerners may offer an explanation for the recent increase in ICC among younger adults [55]. 

### 4.4. Liver and Biliary Tract Malignancies: Current Detection Measures and Screening Guidelines

The American Association for the Study of Liver Diseases (AASLD) recommends HCC screening for all adults with liver cirrhosis to decrease mortality. The AASLD advises liver ultrasound (US) every 6 months for HCC screening with additional testing of serum alpha-fetoprotein (AFP). CT and MRI are not recommended for HCC surveillance in individuals with cirrhosis due to a lack of data on the efficacy and cost-effectiveness of these modalities [84,85]. 

A study performed at the Mayo Clinic demonstrated that annual screening with serum CA 19-9 and cross-sectional abdominal imaging for PSC patients helped identify cancer, which may help improve survival through earlier disease discovery [86]. The AASLD recommends yearly MRI/MRCI plus the consideration of CA 19-9 for the surveillance of ICC and gallbladder carcinoma [87]. In high-prevalence areas of liver fluke infection, such as Thailand, one recent study demonstrated that screening ultrasound led to the diagnosis of ICC at an earlier stage [118,119]. 

## 5. Pancreatic Cancer

### 5.1. Pancreatic Cancer: Introduction

Pancreatic ductal adenocarcinoma (PDAC) is the most common form of pancreatic cancer and accounts for more than 90% of all cases [5]. PDAC typically arises from pancreatic ductal cells, often originating from pancreatic intraepithelial neoplasia (PanIN), and less frequently from intraductal papillary mucinous neoplasms (IPMNs) [120]. 

According to GLOBOCAN, in 2020, there were 496,000 new cases of PDAC, almost matched by the number of deaths (466,000), positioning it as the seventh leading cause of cancer-related death [8]. Globally, both incidence and mortality rates witnessed a 55% and 53% increase, respectively, from 1992 to 2017, and have risen across all age groups [10]. The highest incidence rates were documented in North America, Europe, and Australia [22]. On the other hand, the lowest incidence rates are found in Africa and South-Central Asia [23]. Incidence rates are higher in men compared to women, with a reported global incidence of 5.7 per 100,000 for men and 4.1 per 100,000 for women [8,14]. It is estimated that PDAC will be the second leading cause of malignancy-related mortality in the US by 2030 [121,122]. 

United States data from 2001 to 2015 indicate a higher incidence of PDAC among the Black population across every age group in comparison to the White population [19]. The overall incidence rates were 19.4 per 100,000 for the White population and 24.7 per 100,000 for the Black population. A similar pattern was noted in mortality rates [19]. Among White patients, incidence increased across all age groups from 2001 to 2015, with the most significant increases observed in the younger age groups (<50 years). Among Black patients, there were noteworthy incidence increases in most age groups, except for those aged 40–49 and those over 80 years [19]. 

Pancreatic neuroendocrine tumors (pNETs), arising from the neuroendocrine system, are a rare type of neoplasm. The onset of this type of pancreatic cancer is subtle, and the characteristics of the disease can change with the stage. While some individuals with pNETs are asymptomatic, other groups have issues related to the tumor mass effect or hormone synthesis. Overall, the pNET prognosis is better than that of pancreatic ductal adenocarcinoma [123]. While very uncommon, the incidence of pNETs has been increasing [124]. According to a SEER database analysis, the age-adjusted incidence rate of pNET increased from 0.2 per 100,000 in 1975 to 1.5 per 100,000 in 2017. Notably, a drastic increase in pNET incidence occurred from 2008 to 2013, with the incidence jumping from 0.6 per 100,000 to 1.3 per 100,000. These increases in incidence have occurred across all age brackets, with the most drastic increases noted in those over 65 years old [124]. 

### 5.2. Pancreatic Cancer: Incidence in Young Adults

Early-onset pancreatic cancer (EOPC) remains relatively uncommon, but recent research indicates a rise in incidence. An analysis of the SEER database from 1995 to 2014 revealed that the average APC in pancreatic cancer incidence in the 45 to 49 age group was +0.77% (95% CI, 0.57–0.98), and it was +4.34% (95% CI, 3.19–5.50) in the 25-to-29-year-old age group [32]. A similar APC (+3.4%) in incidence for very early onset (20–39 years at diagnosis) was seen in a separate analysis from 2000 to 2019 [33]. 

Another analysis of SEER from 2004 to 2016 demonstrated a consistent rise in the annual age-adjusted incidence rates (AAIRs) of EOPC across all sexes and racial groups. Additionally, while the AAIR was higher in men, the rate of increase was more rapid among women. Among racial groups, African Americans exhibited the highest AAIR, followed by non-Hispanic White individuals, and Hispanic patients displayed the lowest AAIR, albeit with the fastest increase. The analysis further unveiled that the incidence of all stages of the disease is on the rise, suggesting that the escalation in incidence rates may not primarily stem from the detection of early-stage cases [34]. 

The prognosis for PDAC is grim, having one of the lowest 5-year relative survival rates among all cancer types. There has been a modest improvement in 5-year relative survival rates, increasing from 9% in 2011 to 13% in 2024 [23,125]. However, the overall PDAC outcomes remain poor [23]. Additionally, an analysis of the SEER database from 2004 to 2018 found that EOPC was linked to higher 5-year overall survival (6.9% vs. 5.5%) compared to late-onset PC [126].

### 5.3. Pancreatic Cancer: Risk Factors

A high incidence of PDAC is observed in hereditary syndromes, including familial atypical multiple mole melanoma syndrome (FAMM), Lynch syndrome (hereditary nonpolyposis colorectal carcinoma), Peutz–Jeghers syndrome (PJS), hereditary breast and ovarian cancer syndrome (HBOC), familial adenomatous polyposis (FAP), and hereditary pancreatitis. Moreover, familial PDAC is regarded as a distinct clinical entity that is independent of other known familial syndromes, in which individuals have a significant family history of PDAC, typically involving two or more first-degree relatives [71]. 

Approximately 5–10% of EOPC cases are associated with familial PDAC. A US study involving 826 patients reported that the mean age at diagnosis among familial PDAC patients was 57.6 years, decreased as compared to the sporadic group’s mean age of 61 years. Notably, the familial group exhibited a significantly higher proportion of patients diagnosed at age < 50 years than the sporadic group (36.7% vs. 18.3%; *p* = 0.017) [72]. A more recent study confirmed that a family history of PDAC in a first-degree relative correlated with an increased risk of both EOPC (OR 2.53, 95% CI 1.77–3.61) and very early-onset PDAC (VEOPC), showing a similar risk elevation in both categories [56]. Research indicates that EOPC patients have a notably higher prevalence of germline mutations, including *ATM*, *APC*, *BRCA1*, *BRCA2*, *CDKN2A*, *MLH1*, *MSH2*, *MSH6*, *PMS2*, *PALB2*, *STK11*, and *PRSS1*, with *BRCA1* and *BRCA2* mutations being the most common for early-onset cases [73]. 

Other contributors to EOPC include environmental factors. A comprehensive case–control analysis classifying cases at ages below 60 years as EOPC (n = 1954) and under 45 years as VEOPC (n = 226) displayed differences and similarities between risk factors for both age groups. The findings indicated that consuming more than two standard drinks per day was associated with a higher risk of pancreatic cancer across all age groups, with a stronger effect seen in the VEOPC group. Cigarette smoking was also linked to an increased risk of pancreatic cancer in all age brackets, with a particularly heightened risk for EOPC, but not for VEOPC. Furthermore, diabetes mellitus was found to be associated with an elevated risk of EOPC but not of VEOPC. Obesity (BMI ≥ 30) followed a comparable pattern, displaying an increased risk only in EOPC cases [56]. When comparing risk factors between EOPC and late-onset patients, another study revealed similar findings. Based on the findings, there were no notable disparities observed in terms of gender distribution, medical conditions, and alcohol consumption between the two groups of patients. However, a significant difference was noted in the prevalence of current smokers, with a higher proportion among EOPC patients (56%) compared to the older patient group (28%) (*p* = 0.001). Furthermore, it was observed that EOPC patients initiated smoking at a younger average age (19.8 years, 95% CI 16.7–22.9) compared to the older patients (26.1 years, 95% CI 24.2–28) (*p* = 0.001) [57].

### 5.4. Pancreatic Cancer: Screening Guidelines and Detection

In the screening process for pancreatic cancer, primary imaging tests include endoscopic ultrasonography (EUS), magnetic resonance imaging with magnetic resonance cholangiopancreatography (MRI/MRCP), and computed tomography (CT) with specialized pancreatic protocols. While PDAC screening is not recommended for average-risk patients, screening is a consideration for high-risk populations and associated with early detection [88].

The International Cancer of the Pancreatic Screening (CAPS) consortium represents the only international effort to provide screening guidelines for individuals at risk of pancreatic cancer based on genetic mutation status and family history. Screening is recommended for patients who are carriers of STK11 or CDKN2A mutations, as well as those with a family history and germline *BRCA2*, *BRCA1*, *PALB2*, *ATM*, *MLH1*, *MSH2*, or *MSH6* gene mutations [88,127,128]. Lastly, individuals from familial pancreatic cancer kindreds are also included if they have at least two affected genetically related relatives. The initiation age of surveillance varies and is influenced by the specific genetic mutation and family history. For familial pancreatic cancer without known mutations, screening starts at age 50, or 10 years younger than the age at cancer diagnosis for the youngest affected relative. Mutation carriers have tailored starting ages (e.g., *PJS* at age 40, *CDKN2A* at age 40, *BRCA2*, *ATM*, *PALB2*, *BRCA1*, *MLH1*/*MSH2*, *MSH6* at age 45 or 50). The National Comprehensive Cancer Network (NCCN) guidelines slightly differ from these in that they recommend PJS carriers to begin screening at ages 30–35, and *BRCA2*, *ATM*, *PALB2*, *BRCA1*, *MLH1*/*MSH2*, *MSH6* mutation carriers at age 50 [99]. These efforts prioritize early detection and aim to improve outcomes by identifying pancreatic cancer at its earliest stages. Additionally, the guidelines emphasize the importance of further research, which includes the identification of models for addressing other established risk factors, such as diabetes and markers of metabolic syndrome, smoking habits, a family history of other cancers, gene variants revealed through genome-wide meta-analysis, and predictive biomarkers [88,99]. 

## 6. Colorectal Cancer

### 6.1. Colorectal Cancer: Introduction

Colorectal cancer (CRC) is the third most common cause of cancer-related death and the third most frequently diagnosed cancer in the US. Over 90% of cases are adenocarcinomas, and the remaining 10% include neuroendocrine, squamous cell, adenosquamous, spindle cell, and undifferentiated carcinomas [6]. Data from the SEER program and the Centers for Disease Control (CDC) National Program of Cancer Registries revealed that the US incidence of CRC was 33% higher in men than in women from 2015 to 2019 [15]. The global burden of CRC is estimated to reach over 3.2 million new cases and 1.6 million deaths by the year 2040. An analysis of the GLOBOCAN database in the year 2020 demonstrated that the CRC incidence was highest in Australia/New Zealand and European regions and lowest in several African regions and Southern Asia. Similar results were shown for mortality rates, which were found to be the highest in Eastern Europe and the lowest in Southern Asia [11]. 

The 5-year survival rate for colorectal cancer has improved in recent years. Previously 50% in the 1970s, 5-year CRC survival is now 65%, according to one study analyzing data from 2012 to 2018. This decreased mortality can be attributed to enhancements in screening, imaging, therapy, and surgical techniques [15]. However, improvements in CRC screening uptake have not been even across racial and ethnic groups [129]. Black patients still have higher rates of colorectal cancer mortality within the US [20]. For metastatic CRC, non-Hispanic White patients saw an increase in 5-year survival rates from 9.8% to 15.7% when comparing 1992–1997 to 2004–2009. However, for these same time periods, Black patients saw an increase in 5-year survival from 8.6% to 9.8%. Hence, Black patients may not be benefiting from advances in screening and treatment [130].

### 6.2. Colorectal Cancer: Incidence in Young Adults

One study from the German Center for Cancer Registry Data found EO-CRC to constitute over 5% of all CRC cases and to be steadily increasing over time [35]. Another American study analyzing SEER data from 1975 to 2003 found similar results, with an increase in EO-CRC incidence in younger populations. With current trends, the incidence of colon and rectal cancers may increase by 90.0% and 124.2%, respectively, for individuals in the 20–34-year age group [36]. Similar trends of an increase in EO-CRC incidence have also been noted in many other countries, including Australia, Canada, Denmark, Korea, New Zealand, Slovenia, Sweden, and the UK [37,38,39]. 

### 6.3. Risk Factors/Etiologies of Early-Onset Colorectal Cancer

Lower dietary fiber intake, heavy alcohol use, greater red meat consumption, lack of regular NSAID use, and lower educational level are all non-genetic risk factors for EO-CRC. Other variables also demonstrated an association with early-onset colorectal cancer, including having a history of diabetes and a low intake of folate, fiber, and calcium. Several risk factors for CRC at an older age—but not necessarily EO-CRC—included smoking, an increased BMI, and a lack of aspirin use [58,131]. One population-based case–control study conducted in Ontario, Canada, from 2018 to 2019 found that a higher consumption of sugary beverages, a history of CRC in a first- or second-degree family member, and an increasingly Westernized diet increased the risk of EO-CRC. On the contrary, greater calcium supplement use and a previous diagnosis of allergies or asthma were less likely to be associated with EO-CRC [59]. 

Around 15–20% of patients with CRC found before the age of 50 have a germline mutation that is linked to the malignancy [74,75]. Germline variants with high penetrance are detected in up to 1 in 10 cases of CRC [76]. Hereditary CRC syndromes include familial adenomatous polyposis syndrome (FAP), Lynch syndrome, and hamartomatous polyposis syndromes. FAP is associated with the APC gene mutation (autosomal dominant inheritance) and MUTYH (autosomal recessive inheritance). Lynch syndrome is associated with abnormalities in the *MLH1*, *MSH2*, *MSH6*, and *PMS2* genes. Hereditary hamartomatous syndromes include Peutz–Jeghers syndrome (PJS, with mutations in LKB1/STK11), PTEN-hamartoma tumor syndrome (PHTS, with mutations in PTEN), and juvenile polyposis syndrome (JPS, with mutations in BMPR1A or SMAD4) [77]. Lynch syndrome is the most common hereditary form of CRC and is implicated in almost 20% of people diagnosed with EO-CRC. In one study, 26% of EO-CRC patients had a first-degree family member also previously diagnosed with CRC. Hence, genetic testing is highly recommended in all patients found to have EO-CRC [75]. 

### 6.4. Screening for Colorectal Cancer/Current Detection Measures

Common methods for non-invasive colon cancer detection include guaiac fecal occult blood testing (gFOB), fecal immunochemical testing (FIT), and multitarget stool DNA testing (MT-sDNA, marketed as Cologard). While MT-sDNA has a higher sensitivity than FIT and gFOB, it has up to 3 times more false positive results than FIT [89,90,91]. A randomized population-based study concluded that FIT screening had superior participation and detection rates and should be preferred over gFOB screening if MT-sDNA testing is unavailable [89,92]. The gold standard of CRC screening is colonoscopy. Obtaining an individual’s family history is imperative, as high-risk persons may qualify for earlier-age CRC screening [132]. A study involving 88,902 participants over 22 years found that negative colonoscopy is strongly associated with a significantly reduced risk of proximal and distal CRC [93]. Colonoscopy is also used to diagnose CRC or to find and remove colorectal polyps following a positive non-invasive test [89,90]. Another direct visualization test includes flexible sigmoidoscopy; however, it does not include the visualization of the entire colon and could lead to missed lesions. CT colonography uses serial radiographic images to visualize the entire colon. Notably, abnormalities noted on flexible sigmoidoscopy or CT colonography typically require a full colonoscopy for a more detailed examination [133]. A blood-based CRC screening test was recently FDA-approved (PCR-based Epi proColon), which is intended for use by individuals unable or unwilling to complete other screening modalities. This blood test detects methylated DNA from CRC cells but is only 68.2% sensitive for cancer discovery compared to colonoscopy [94]. 

The incidence of colorectal adenocarcinoma has been rising in recent years, with 15% more cases diagnosed among individuals between the ages of 40 and 49 years from 2014 to 2016 compared to 2000–2002. Therefore, the United States Preventative Service Task Force (USPSTF) currently recommends screening for colorectal cancer in all individuals starting at 45 years of age, a change from its prior recommendation of starting at age 50. Appropriate intervals and modalities include a colonoscopy every 10 years, flexible sigmoidoscopy every 10 years combined with FIT every year, flexible sigmoidoscopy every 5 years, CT colonography every 5 years, MT-sDNA every 1 to 3 years, or high-sensitivity gFOB or FIT.

There are variations in CRC screening internationally. For example, the Canadian Task Force on Preventative Health Care does not encourage the use of colonoscopy for CRC screening purposes in healthy individuals without a family history of CRC, though this is a weak recommendation based on low-quality evidence. Instead, they recommend Canadians receive gFOBT or FIT in two-year intervals or a flexible sigmoidoscopy every 10 years. Notably, these guidelines cannot be applied to individuals with a history of polyps [134]. Australian national guidelines recommend those without a family history of CRC to be screened with gFOB every 2 years between the ages of 50 and 74 [135]. The European Commission in 2022 recommended annual FIT as a first-line test for people aged 50–74, with colonoscopy referrals exclusively for those with positive stool testing [136]. Asian countries such as South Korea recommend using colonoscopy, gFOB, or FIT for CRC screening. Chinese recommendations include gFOB or FIT every 3 years, and Saudi Arabia guidelines encourage colonoscopy every 10 years [90,137].

One study aimed to identify ways in which EO-CRC could be diagnosed earlier in those aged 40 to 49 years. Gupta et al. found that roughly one-quarter of all individuals diagnosed with EO-CRC in this age group met the criteria for early screening due to family history. Another population-based study was designed to determine what proportion of EO-CRC cases could have been prevented if current screening guidelines were followed. The findings of this study suggested that if current screening guidelines were upheld, 52% of EO-CRC cases could have been identified sooner, and 16% could have potentially been prevented [138]. Hence, using proper screening strategies could help with the prevention or sooner detection of EO-CRC in select populations [138]. Having a first-degree relative with colorectal cancer confers a 2-times greater likelihood of developing this disease. Therefore, the US Multisociaty Task Force on CRC (USMSRF) recommends that high-risk populations commence screening through colonoscopy by age 40 at the latest. Screening can start earlier if needed and is recommended to begin 10 years prior to the age at diagnosis of the first-degree relative if this would lead to screening before age 40 [139].

Patients who carry hereditary CRC syndromes are recommended to begin screening for CRC even earlier. Individuals with FAP are recommended to begin screening at the age of 10–12, those affected by Lynch syndrome are recommended to begin screening at the age of 20–25, and patients with biallelic MUTYH-associated polyposis are recommended to begin screening at the age of 25–30 [100,101,102]. Lastly, individuals with either Juvenile polyposis or Peutz–Jeghers are recommended to initiate screening at 15 years [100].

## 7. Conclusions

Colorectal, gastric, pancreatic, and biliary tract malignancies have seen a rise in incidence among young adults over the past thirty years [140]. Early screening for high-risk populations, such as those with an established family history and/or genetic predisposition, can aid in cancer prevention and decrease the morbidity of cancer through earlier detection and treatment [139]. Individuals with obesity, Crohn’s or ulcerative colitis, a sedentary lifestyle, diabetes, a history of tobacco use, and unhealthy dietary habits are at particular risk for CRC [141]. Alcohol and smoking are well-established risk factors for EOPC, and PSC places individuals at high risk for biliary malignancy [53,56,57]. CDH1 abnormalities and PJS are known genetic risk factors for early-onset gastric cancer [64,66]. Lifestyle factors, such as obesity or morbid obesity, dramatically increase the risk of early-onset esophageal adenocarcinoma. Although there are many established genetic and environmental susceptibilities for early-onset gastrointestinal carcinogenesis, future population-based studies may help identify additional risk factors to permit further tailoring of cancer prevention and early detection strategies.

## Figures and Tables

**Table 1:** Overview of gastrointestinal and liver cancers: global population trends.

	Esophageal Cancer	Gastric Cancer	Liver Malignancies	Pancreatic Cancer	Colorectal Cancer
Type	Esophageal adenocarcinoma (EAC), squamous cell carcinoma (SCC) [2]	Predominantly adenocarcinoma - Cardia - Non-cardia [3,4]	Hepatocellular carcinoma (HCC), intrahepatic cholangiocarcinoma (ICC)	Predominantly pancreatic ductal adenocarcinoma [5]	Predominantly adenocarcinoma [6]
Global incidence	EAC: Increasing SCC: Decreasing [2]	Increasing [7]	HCC: Decreasing ICC: Increasing [8,9]	Increasing [10]	Increasing [11]
Sex Predilections	Male > Female [12]	Male > Female [8,13]	Male > Female [8]	Male > Female [8,14]	Male > Female [15]
Racial Predominance	EAC: White SCC: Black [16]	Black and Hispanic [13,17]	HCC: Asian American/Pacific Islander, Black ICC: Asian American/Pacific Islander [18]	Black [19]	Black [20]
Regional Differences	Highest: Eastern Asia and Southern and Eastern Africa Lowest: Western Africa and Central America [2]	Highest: Asia Lowest: North America and Africa [8]	HCC Highest: Eastern Asia, Northern Africa, Eastern Europe Lowest: South-Central Asia ICC Highest: South-Eastern Asia, Eastern Asia, and Northern Europe Lowest: South-Central Asia [21]	Highest: North America, Europe, and Australia Lowest: African regions and South-Central Asia [22,23]	Highest: Australia/New Zealand Lowest: African regions and Southern Asia [11]

**Table 2:** Characteristics of early-onset gastrointestinal malignancies.

	Esophageal Cancer	Gastric Cancer	Liver Malignancies	Pancreatic Cancer	Colorectal Cancer
Trends of Early-Onset Cancer	EAC: Plateau or decreasing [17,24,25] SCC: Decreasing [25]	Increasing [26,27,28]	HCC: Decreasing [29,30] ICC: Increasing [30,31]	Increasing [32,33,34]	Increasing [35,36,37,38,39]
Risks for early-onset cancer: environmental	EAC: Barrett’s esophagus, obesity [40,41] SCC: Oral hygiene, tobacco smoking [42]	*H. pylori* infection, obesity, heavy alcohol use [43,44,45,46,47,48,49,50]	HCC: Chronic hepatitis B infection, tobacco smoking [51,52] ICC: Primary sclerosing cholangitis, parasitic infections, hepatolithiasis [53,54,55]	Heavy alcohol use, tobacco smoking, diabetes, obesity [56,57]	Lower dietary fiber intake, heavy alcohol use, greater red meat consumption, lack of regular nonsteroidal anti-inflammatory drug use, and lower educational level [58,59]
Risks for early-onset cancer: genetic, familial	Familial Barrett’s esophagus [60]	Family history, CDH1 germline mutation, Lynch syndrome, juvenile polyposis syndrome, Peutz–Jeghers syndrome [61,62,63,64,65,66,67]	HCC: Family history of HCC, family history of hepatitis B infection [68,69] ICC: Congenital disorders of biliary tract [70]	Family history of pancreatic ductal adenocarcinoma, multiple germline mutations (*BRCA1/2*, *PALB2*, *APC ATM*, *CDKN2A*, *MLH1*, *MSH2*, *MSH6*, *PMS2*, *EPCAM STK11*, *PRSS1*) [71,72,73]	Lynch syndrome, familial adenomatous polyposis, juvenile polyposis syndrome, Peutz–Jeghers syndrome, PTEN-hamartoma [74,75,76,77]

EAC: esophageal adenocarcinoma; SCC: squamous cell carcinoma; HCC: Hepatocellular carcinoma; ICC: Intrahepatic cholangiocarcinoma.

**Table 3:** Screening methods and recommendations for general population.

	Esophageal Cancer	Gastric Cancer	Liver Malignancies	Pancreatic Cancer	Colorectal Cancer (CRC)
Primary screening methods	Esophagogastroduodenoscopy (EGD), unsedated transnasal endoscopy, cytosponge [78,79,80]	EGD, contrast (barium) radiographic screening [81,82,83]	Liver ultrasound, serum alpha-fetoprotein for hepatocellular carcinoma [84,85,86], MRI/MRCP, serum CA 19-9 for ICC [87]	Endoscopic ultrasonography, MRI/MRCP, computed tomography [88]	Fecal immunochemical testing, multitarget stool DNA testing, colonoscopy, PCR-based detection of methylated DNA (Epi pro colon) [89,90,91,92,93,94]
Current screening guidelines and recommendations	EAC: Barrett‘s Esophagus Screening guidelines [78] SCC: No current guidelines [95]	US: No screening guidelines, though recommended screening for those with hereditary gastric cancer syndromes [81,82] East Asia: EGD screening programs in Japan and Korea [82,96,97,98]	HCC: Recommended for all patients with cirrhosis [84,85] ICC: Yearly MRI/MRCP with or without serum CA 19-9 in individuals with PSC [87]	Recommended for carriers of *STK11*, *PRSS1*, or *CDKN2A* mutations, those with a family history and germline *BRCA2*, *BRCA1*, *PALB2*, *ATM*, *p53*, *MLH1*, *MSH2*, or *MSH6* gene mutations [88,99], and those with two or more relatives (from the same side of the family) who developed PDAC	Early screening is recommended for patients carrying hereditary CRC syndromes, including familial adenomatous polyposis, Lynch syndrome, and biallelic MUTYH-associated polyposis, in addition to routine screening for sporadic CRC [100,101,102]
Recommended age of screening initiation	EAC: No specific age of initiation SCC: No current guidelines	East Asia: ≥40 years (Korea), ≥50 years (Japan) [82,96,97,98]	No specific age of initiation	Peutz–Jeghers syndrome ≥ 40 years, CDKN2A ≥ 40 years, germline (*BRCA2*, *ATM*, *PALB2*, *BRCA1*, *MLH1*/*MSH2*) ≥ 45–50 years, familial PDAC (≥50 or 10 years younger than age at diagnosis of youngest relative) [88,99]	Sporadic ≥ 45 (US), familial adenomatous polyposis ≥ 10–15 years, Lynch syndrome ≥ 20–25 years, juvenile polyposis syndrome and Peutz–Jeghers syndrome ≥ 15 years, PTEN-hamartoma tumor syndrome ≥ 35 years [100,101,102]

EAC: esophageal adenocarcinoma; SCC: squamous cell carcinoma; PSC: primary sclerosing cholangitis; MRI/MRCP: magnetic resonance imaging/magnetic resonance cholangiopancreatography; PDAC: pancreatic ductal adenocarcinoma; HCC: Hepatocellular carcinoma; ICC: Intrahepatic cholangiocarcinoma.
